# An Evolutionary Weakness: Can We Exploit Parasite–Bacteria Symbiosis?

**DOI:** 10.3390/pathogens15101076

**Published:** 2026-10-10

**Authors:** Stephanie Berry, Felix Theml, Terry K. Smith

**Affiliations:** Biomedical Sciences Research Complex, School of Biology, University of St Andrews, North Haugh, St Andrews KY16 9ST, UK

**Keywords:** endosymbiosis, *Wolbachia*, filarial nematodes, protozoan parasites, host–microbe interactions, parasite virulence

## Abstract

Exploiting the endosymbiotic relationship between some parasites and bacteria may be an exciting therapeutic strategy. The obligate relationship between *Wolbachia* and nematodes represents a successful model, where the disruption of bacterial metabolism leads to parasite death, although challenges in applying novel drugs remain. Obligate endosymbiosis is rare in protozoa, limiting its broader application. Instead, facultative associations, such as those between *Trichomonas vaginalis* and *Mycoplasma hominis* and *Entamoeba* spp. and gut bacteria, demonstrate complex effects on parasite virulence and treatment outcomes. Successfully exploiting symbiosis depends on the stability and necessity of the symbiotic relationship, as well as the accessibility of the bacterial partner.

## 1. Symbiotic Relationships Between Parasites and Bacteria

Symbiosis refers to a close and long-term interaction between two organisms, which may be mutually beneficial or confer an advantage to one partner. It is a fundamental and ancient concept in biology; the emergence of the first eukaryotic cells is thought to have occurred at least 1.7 billion years ago through an endosymbiotic event involving an archaeal host and an engulfed bacterial cell [1]. Bacterial symbiosis is widespread across all domains of life, occurring in associations with other bacteria, protozoa and multicellular organisms [2]. These interactions are of particular interest in the context of infectious disease, as symbiotic relationships can directly influence pathogen survival, transmission and virulence. Pathogens that rely upon symbiosis for essential functions may present novel targets for therapeutic intervention.

Parasites are eukaryotic organisms that survive by exploiting a host [3]. Human parasitic diseases are primarily caused by two groups: protozoa, which are unicellular, and helminths, which are multicellular worms. Parasites can be transmitted by biological vectors, as well as through other mechanisms, such as food/water contamination [3,4]. Many parasites form symbiotic relationships with bacteria, which can range from obligate interactions, where the removal of the symbiont is lethal, to facultative associations that enhance parasite fitness or virulence [5]. Understanding these relationships is increasingly important in drug development, as they can provide novel therapeutic targets, but they also add new levels of complexity. This review will evaluate examples of recent advances in targeting parasite-associated bacterial symbioses, focusing on their potential and limitations in the development of antiparasitic therapies.

## 2. Targeting Obligate Bacterial Endosymbionts

### Wolbachia and Nematodes

*Wolbachia* is a genus of α-proteobacteria that is one of the most widespread groups of endosymbionts, infecting a broad range of arthropods and nematodes [6]. In arthropod hosts, *Wolbachia* can manipulate host reproduction, such as through cytoplasmic incompatibility, where infected males can only successfully reproduce with infected females. This gives infected females an advantage, allowing for the rapid spread of bacteria [6,7]. This capacity has been successfully exploited in vector control programmes, particularly in mosquito populations, where the introduction of *Wolbachia* into mosquito populations reduces *Plasmodium* transmission indirectly by altering mosquito biology, thereby limiting the ability of the parasite to develop and be transmitted [7]. However, in filarial nematodes, *Wolbachia* plays a different role, forming an obligate mutualistic relationship that is essential for host survival and reproduction.

**Figure 1:**
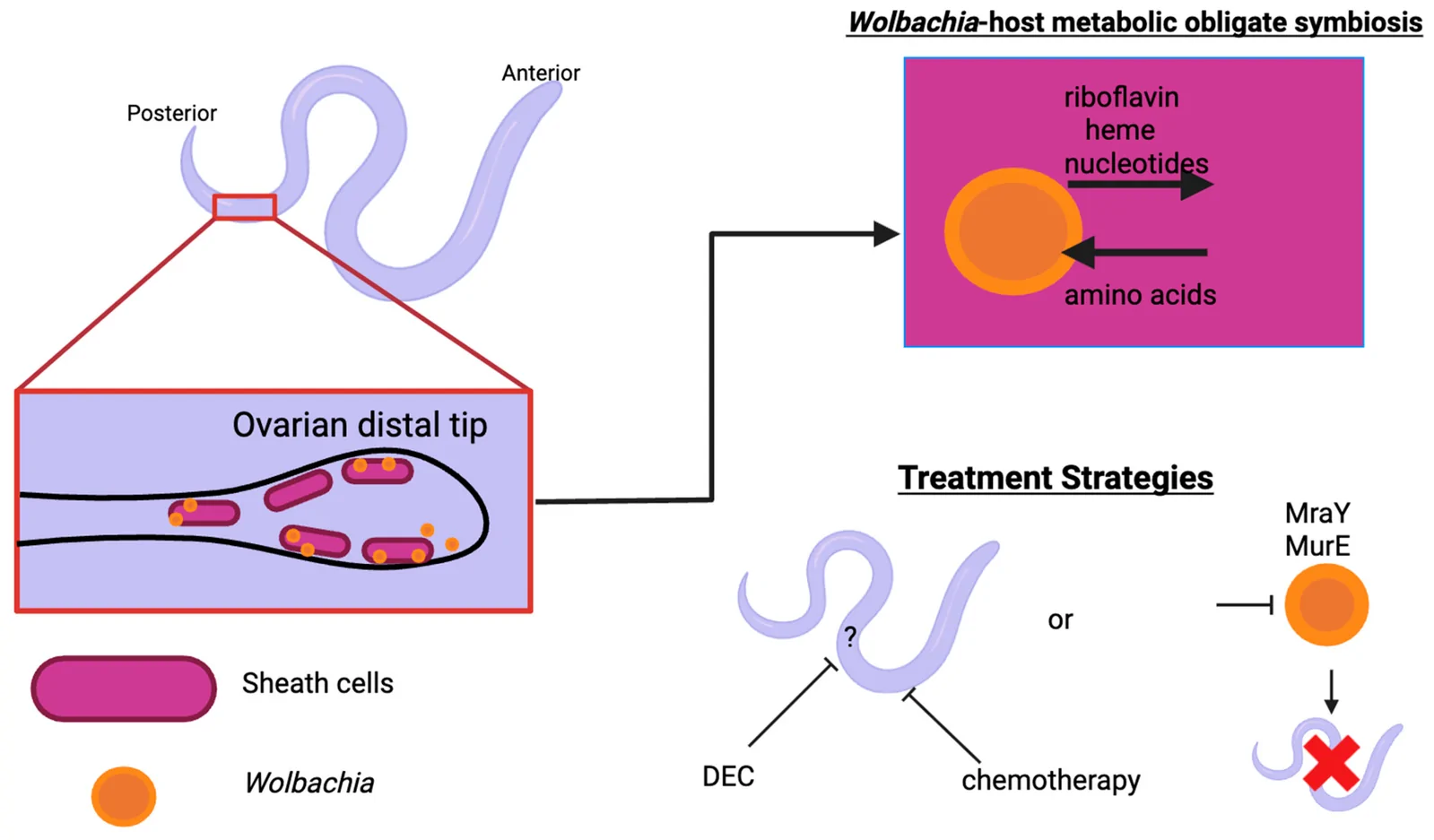
The treatment rationale and strategies for filarial nematodes. *Wolbachia* is an obligate parasite that resides in the sheath cells of filarial nematodes, which promotes onward transmission to offspring during nematode reproduction. *Wolbachia* mediates an obligate relationship through metabolic dependency, where the bacteria and host exchange metabolites for survival. Therefore, drug targets, such as MraY and MurE, provide alternative treatment strategies compared to diethylcarbamazine (DEC) and chemotherapy, which are preferentially used. This figure was generated using biorender.com.

*Wolbachia* obligate endosymbiosis is evident in nematodes responsible for neglected tropical diseases, such as *Onchocerca volvulus* (onchocerciasis), *Dirofilaria* spp. (hongkongensis) and *Wucheria bancrofti* and *Brugia* spp. (lymphatic filariasis) [8,9,10]. Within these parasites, *Wolbachia* localises to specific tissues, including the hypodermal lateral cords and the female reproductive system, where it is closely associated with developing oocytes [11]. Structural studies have demonstrated the presence of *Wolbachia* within sheath cells, with cytoskeletal-like connections linking these cells to oocytes, suggesting a mechanism for efficient vertical transmission (Figure 1) [11]. At the metabolic level, symbiosis is characterised by reciprocal dependency (Figure 1). *Wolbachia* supplies essential metabolites, including riboflavin, haem and nucleotides, which the nematode is unable to synthesise independently [12]. In return, the bacterium relies on the host for amino acids and other key nutrients. The functional importance of this relationship is highlighted when *Wolbachia* is depleted using antibiotics, which results in the sterility and death of the nematode [13]. While the precise mechanisms underlying this infertility remain unknown, it is possible that the disruption of metabolite provision, such as haem biosynthesis, impairs oocyte development and embryogenesis.

Current treatments for filarial diseases include chemotherapy and diethylcarbamazine (DEC), which is not effective in all cases and in unsuitable when contraindications are present, such as a second filarial disease as highlighted by WHO and CDC [14]. The obligate nature of the interaction between these nematodes and *Wolbachia* makes *Wolbachia* an attractive drug target for filarial diseases. However, targeting an intracellular endosymbiont poses significant pharmacological challenges. Drug candidates must penetrate multiple biological barriers, including host tissue, nematode cuticle and cellular structures and the double membrane of the Gram-negative bacterium. This complexity is reflected in current treatment strategies, where antibiotics such as doxycycline, minocycline and rifampicin are effective but require prolonged administration, limiting their scalability in mass drug administration programmes [15].

Recent research has focused on identifying novel *Wolbachia*-specific drug targets, which may improve selectivity. Enzymes involved in peptidoglycan biosynthesis, such as MraY and MurE, have emerged as promising candidates [16,17,18]. Targeting these essential pathways may therefore provide selective antibacterial effects, limiting the risk of adverse side effects. In addition, natural products such as corallopyronin A, which inhibits bacterial DNA-dependent RNA polymerase, have shown considerable promise [17]. Corrallopyronin A demonstrates effective penetration into nematode tissues and a shorter treatment course compared to conventional antibiotics, addressing a key limitation of current therapies [11].

Despite these advances, challenges remain. The recent failure of candidates such as AWZ1066S in early clinical trials highlights the difficulty of translating in vitro efficacy into safe and effective treatments, particularly if the molecular target is not well defined [18,19]. This reveals a broader issue in endosymbiont-targeted therapy: a detailed mechanistic understanding of both bacterial and host biology is essential to minimise off-target effects and optimise drug design.

Overall, the *Wolbachia*–filarial nematode system provides an example of how obligate endosymbiosis can be exploited therapeutically; however, it also illustrates the challenges of targeting intracellular symbiosis, where biological complexity and drug delivery barriers must be carefully navigated to achieve clinical success.

## 3. Obligate Bacterial Endosymbionts and Protozoan Parasites

**Figure 2:**
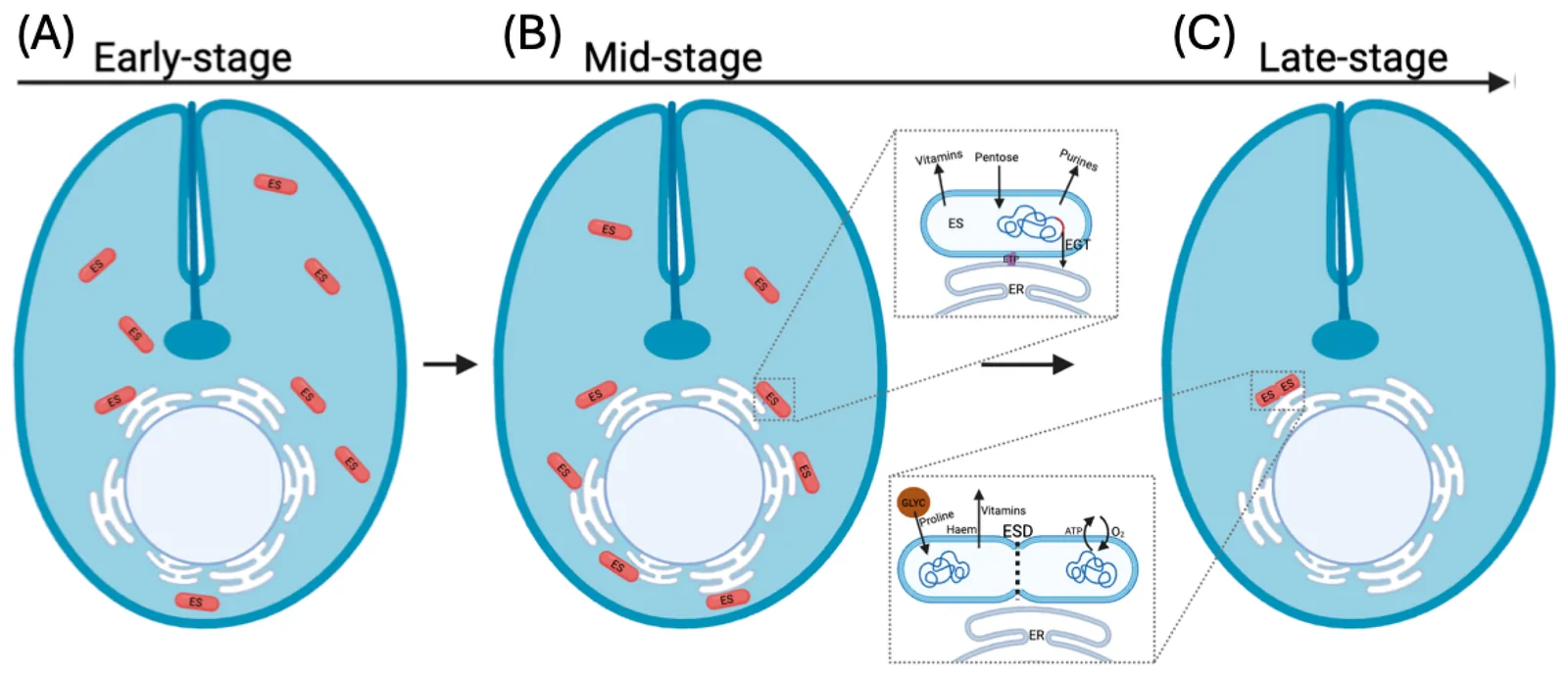
Endosymbiont evolution in trypanosomatids. The evolution of endosymbiosis in trypanosomatids is a gradual process. (**A**) In early-stage endosymbiosis, a prokaryote with beneficial metabolic traits enters trypanosomatids. (**B**) In the mid-stage of endosymbiosis, the metabolic benefit conveyed by the endosymbiont becomes more apparent, and metabolic integration occurs, while the loss of duplicate genes may occur on either side. Some metabolic pathways may become interlinked, requiring enzymes from the host and endosymbiont [21]. (**C**) In late-stage endosymbiosis, the endosymbiont is fully integrated into the host metabolism with active metabolite exchange. ES—endosymbiont; ER—endoplasmic reticulum; EGT—endosymbiont gene transfer; ETP—endosymbiont-targeted protein; GLYC—glycosome; ESD—endosymbiont division site. This figure was created using BioRender.com.

In contrast to the well-characterised obligate endosymbiosis observed in filarial nematodes, such relationships appear to be rare in protozoan parasites. Obligate bacterial endosymbiosis requires the evolution of tightly integrated metabolic interdependence, in which both partners rely on one another for essential cell functions (Figure 2). While this is readily achieved in multicellular hosts that can provide greater cellular complexity and capacity for compartmentalisation, the establishment of similarly stable systems in unicellular protozoa presents additional biological constraints. One key limitation is the reduced cellular complexity of protozoa, which restricts their capacity to compartmentalise and regulate intracellular symbionts [20]. Although some protozoa can harbour bacteria within vacuoles or cytoplasmic niches, maintaining long-term mutualistic relationships without incurring significant fitness costs appears to be more challenging. Experimental studies have demonstrated that while a wide range of protozoa–bacteria associations can form, many result in decreased host fitness, suggesting that stable obligate endosymbiosis represents a relatively rare evolutionary outcome [20].

Despite these constraints, exceptions exist within the trypanosomatids, a group of flagellated protozoa (Table 1). Several species, collectively termed symbiont-harbouring trypanosomatids (SHTs), including *Strigomonas*, *Angomonas* and *Kentomonas* species (one of the newest members of this selective family), maintain obligate endosymbiosis with intracellular bacteria belonging to the genus *Candidatus* Kinetoplastibacterium [21,22,23]. This endosymbiont, *Candidatus Kinetoplastibacterium* sp., found in all but one member of the trypanosomatid sub-family *Strigomonadinae*, was acquired in a single event from a single ancestor around 40–120 million years ago [24,25,26]. It remains elusive whether the beta-proteobacterium endosymbiont was acquired through exposure in an insect gut. Studies to establish what prompted the single rogue member of Strigomonadinae to lose its endosymbiont will provide unique insight into the evolutionary costs associated with the acquisition and maintenance of an endosymbiont. The loss of the endosymbiont is particularly interesting considering that the horizontal transfer of the Strigomonadinae endosymbiont may occur even across species, likely during the co-infection of the same insect host [22].

Ecto-phosphatase activities and specificities were shown to be different in symbiont-bearing and aposymbiotic strains of *Strigomonas culicis*, suggesting that the symbiont influences host protozoan ecto-phosphatase activity and indicating a possible role of this enzyme during mosquito tissue colonisation [27].

The model organism for studying end-stage endosymbiosis in trypanosomatids is *Angomonas deanei* due to its precise control division over its endosymbiont governed by a series of endosymbiont-targeted proteins (ETPs), most notably the dynamin-like protein ETP9. ETP9 is essential in ESD, co-localising with FtsZ, the protein responsible for canonical bacterial cytokinesis [28].

The presence of the endosymbiont is linked with a high degree of metabolic integration [29], with increased O_2_ consumption associated with oxidative phosphorylation [30]. This increased metabolic efficiency is translated to the reduced excretion of fermentation products and greater adaptability to food scarcity. The reverse is observed in aposymbiotic strains, including of other Strigomonadinae like *Strigomona culicis*, resulting in increased fermentation [30,31]. Both studies use similar methods to investigate oxygen consumption, allowing for a comparison of the two species; however, it would be beneficial to investigate the metabolism of *S. culicis* using alternate carbon sources such as a strict proline diet, as was shown for *A. deanei*. The benefits conveyed by the endosymbiont reach beyond oxidative phosphorylation, providing haem, vitamins and amino acids as well as resistance to reactive oxygen species to the host [32,33].

In both *S. culicis* and *A. deanei*, the endosymbiont is closely associated with host glycosomes, due to its dependency on glycosome-derived 2-oxoglutarate for NADPH generation and fructose-1,6-bisphosphate as the energy source for oxidative phosphorylation [23]. Further, the EGT-derived ornithine cyclodeaminase proves to be an outlier to the assumption that proteins gained through EGT must be targeted to the endosymbiont to perform their native function there [34]. Instead, the protein is targeted for the host glycosome where it is responsible for proline production. The novel targeting of the protein can be explained when considering that the endosymbiont lost the ability to produce proline independently and thus is reliant on glycosome-derived host proline. This reliance may have been a major driver in metabolic integration and control over endosymbiont localisation in *A. deanei*.

In fact, many of the key metabolic pathways to de novo form lipids, purines and amino acids have been shown to span both these trypanosomatid hosts and their endosymbiosis for mutual benefit [34] (Table 1).

The monoxenous trypanosomatid *Novymonas esmeraldas*, harbouring a recently acquired endosymbiont *Candidatus Pandoraea novymonadis*, presents a valuable opportunity to study the mid-stages of endosymbiosis [35,36].

This is not the only reason for the scientific interest in *N. esmeraldas*, as the parasite presents a valuable model for the insect stage of the closely related human pathogen *Leishmania major*, sharing strong sequence similarity across several key metabolic enzymes. Interestingly, *N. esmeraldas* is missing genes for important metabolic enzymes including transketolase, the absence of which may be compensated by orthologues carried within the endosymbiont genome [34]. It would be interesting to investigate whether the transketolase gene was lost in *N. esmeraldas* as a consequence of duplicate gene loss related to the endosymbiont or at earlier stages of the species’ evolution. Overall, the endosymbiont becomes more apparent, and metabolic integration occurs, such as through EGT, transferring useful genes to the host, while the loss of duplicate genes may occur on either side. Some metabolic pathways may become interlinked, requiring enzymes from the host and endosymbiont [34,37]. The number of endosymbionts per host cell is not regulated, but early attempts at such regulation are ongoing, including exerting control over the localisation of endosymbionts, via the duplication and neo-functionalisation of existing genes [21]. The endosymbiont is found to be close to the endoplasmic reticulum as it starts to rely on ER-derived metabolites (Figure 2B). This results in the more controlled and thus regular transfer of the endosymbiont to daughter cells.

In many of these examples, the bacterial endosymbiont provides essential metabolic capabilities that are absent in host trypanosomatids, most notably the biosynthesis of haem. This represents a significant divergence from medically important trypanosomatids such as *Trypanosoma brucei* and *Trypanosoma cruzi*, which lack haem biosynthetic pathways and instead scavenge this molecule directly from their human hosts [23].

The metabolic integration observed in SHTs closely parallels that of the *Wolbachia*–nematode system, in that the host becomes dependent on the endosymbiont for essential biochemical functions. However, SHTs are not associated with human disease, limiting their immediate therapeutic significance [23]. Nevertheless, they provide valuable model systems for understanding the evolution and mechanistic basis of obligate endosymbiosis in protozoa. They demonstrate that, under appropriate evolutionary pressures, even unicellular eukaryotes can develop stable, metabolically integrated relationships with bacteria. Current evidence suggests that obligate bacterial endosymbiosis is not a widespread feature of protozoan pathogens, and its broader applicability to protozoan diseases is likely to be limited.

Overall, protozoan–bacterial endosymbiosis illustrates the importance of evolutionary and cellular context in determining the viability of symbiont-targeted therapies. Unlike the exploitable dependency observed in *Wolbachia*-associated nematodes, protozoan systems are less predictably dependent on bacterial partners and therefore are unlikely to be suitable therapeutic targets until identified in human pathogens.

## 4. Targeting Parasite-Associated Bacteria

### Trichomonas vaginalis and Mycoplasma hominis

*Trichomonas vaginalis* is a flagellated protozoan parasite and is the causative agent of trichomoniasis, one of the most prevalent non-virally sexually transmitted diseases (STDs) worldwide, as highlighted by the CDC.

In contrast to the obligate endosymbiosis observed in filarial nematodes, *T. vaginalis* engages in a facultative interaction with the bacterium *Mycoplasma hominis*, a commensal of the human urogenital tract. *M. hominis* is also largely asymptomatic; however complications have been demonstrated to cause bacterial vaginosis, pelvic inflammatory disease, infertility and preterm childbirth [38]. Interestingly, *M. hominis* has been shown to reside intracellularly within *T. vaginalis* in 5 to 89% of isolates, representing a rare example of intracellular symbiosis where both organisms retain independent pathogenic potential [37]. This variation in the prevalence of association between populations suggests that this relationship is not essential, implying both environmental exposure and host–microbe compatibility, in terms of specific strains, which may influence the establishment/longevity of this relationship.

The interaction between *T. vaginalis* and *M. hominis* is relatively well-studied compared to many other pathogen–symbiont relationships and appears to involve both cooperative and antagonistic effects. On one hand, the presence of *M. hominis* enhances parasitic growth, adherence to host epithelial cells and cytotoxicity, thereby increasing the pathogenic potential of *T. vaginalis* [39]. On the other hand, *M. hominis* can stimulate host immune responses, particularly through the induction of type I interferon pathways, such as INFε, which are detrimental to parasite survival [40]. This apparent contradiction highlights a finely balanced interaction, in which *T. vaginalis* must mitigate immune activation while still benefiting from bacterial co-infection. One mechanism by which *T. vaginalis* achieves this balance is through the secretion of extracellular vesicles (EVs), which mediate immune modulation via their cargo, which include factors that downregulate interferon signalling pathways [39,40]. This immune modulation not only promotes parasite survival but may also indirectly support the persistence of *M. hominis*, illustrating how the interaction extends beyond simple coexistence to involve the coordinated manipulation of the host environment. Importantly, the hormonal regulation of INFε, particularly its decline in post-menopausal woman, may provide an explanation for increased susceptibility and severity of infection in older populations [41,42].

Despite its high global burden, it remains classified as a neglected parasitic infection, partly due to its asymptomatic presentation. Clinically, however, infection can be associated with complications, including increased susceptibility to other STDs (including human immunodeficiency virus [HIV]), adverse pregnancy outcomes and infertility [43]. The presence of *T. vaginalis* can also alter the vaginal microbiome, which consists of a complex population in women of child-bearing age, and this ultimately decreases the number of beneficial *Lactobacillus* bacteria and increases harmful anaerobes, leading to a state of microbial imbalance called dysbiosis [40,41]. Standard treatment relies on nitroimidazole drugs, such as metronidazole and tinidazole, although emerging resistance and treatment failures are increasingly reported [42,43,44].

**Figure 3:**
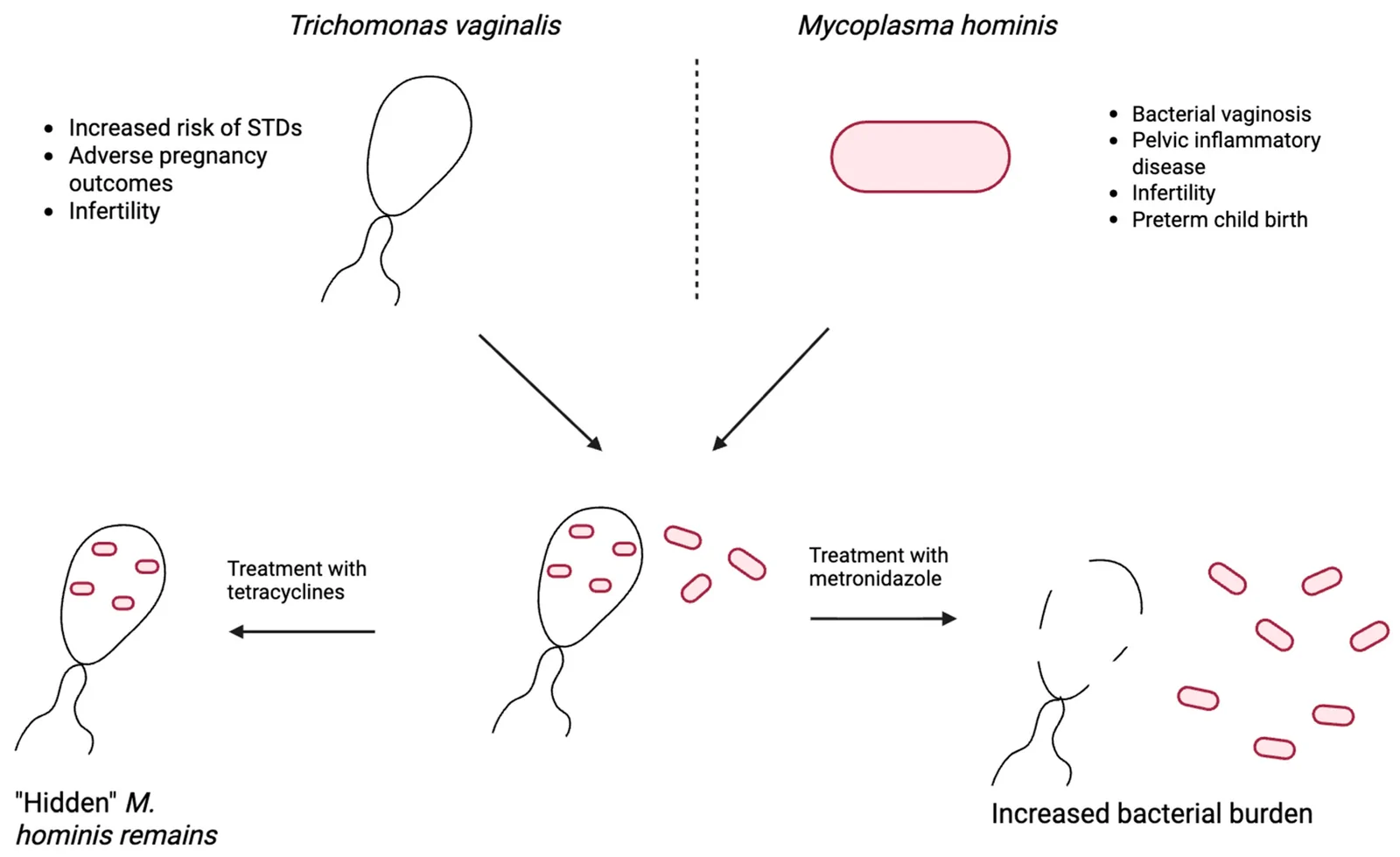
Clinical implications of *Trichomonas vaginalis* and *Mycoplasma hominis* infections. Adverse outcomes are possible in cases of co-infection. Figure generated using biorender.com.

From a therapeutic perspective, this symbiosis presents significant challenges. The treatment of *T. vaginalis* with metronidazole can lead to the release of intracellular *M. hominis* upon parasite lysis, a “Trojan horse” scenario, potentially exacerbating bacterial infection and contributing to adverse outcomes, particularly during pregnancy [43,44]. Furthermore, *M. hominis* lacks a cell wall, rendering it intrinsically resistant to many commonly used antibiotics and limiting treatment options to specific classes, such as tetracyclines and certain fluoroquinolones [45]. This creates a clinical dilemma in which targeting one organism may unintentionally promote the persistence or dissemination of another. Interestingly, some studies have reported that *T. vaginalis* isolates harbouring *M. hominis* exhibit increased sensitivity to metronidazole, although the underlying mechanism remains unclear [46]. This observation further complicates the interaction, suggesting that the presence of the bacterium may simultaneously enhance parasite pathogenicity while increasing susceptibility to treatment. These eventualities are summarised in Figure 3. Such findings reveal the difficulty of predicting treatment outcomes in polymicrobial infections.

Another example of this dilemma is *Acanthamoeba*, which is a free-living amoeba found in soil and water that feeds on environmental bacteria and often infects people through contaminated water coming in contact with skin wounds, the nose and primarily the eyes. However, *Legionella* is able to survive and replicate within *Acanthamoeba* by blocking phagosome–lysosome fusion and forming a specialised *Legionella*-containing vacuole; this in turn increases *Legionella* infectivity by up to 100-fold, making it more invasive to human cells by reprogramming the *Acanthamoeba* transcriptome [47,48].

Overall, the relationship between *T. vaginalis* and *M. hominis* highlights the complexity of parasite-associated bacterial interactions, in which symbiosis is neither strictly beneficial nor detrimental but highly context-dependent. Unlike the obligate and metabolically defined dependency observed in *Wolbachia*–filarial systems, this interaction is more transient and ecologically driven, limiting its suitability as a straightforward drug target. It raises important clinical questions regarding treatment strategies, particularly whether co-infections should be routinely targeted in parallel. This example therefore illustrates that while symbiotic interactions can influence disease progression and treatment response, their therapeutic exploitation requires a nuanced understanding of both microbial and host dynamics.

## 5. *Entamoeba* spp. and *Escherichia coli*

*Entamoeba histolytica* and *Entamoeba dispar* are non-flagellated protozoan parasites that colonise the human intestine and are associated with amoebiasis. While *E. histolytica* is the primary pathogenic species, *E. dispar* has traditionally been considered non-pathogenic; however, its virulence can be influenced by interactions with the gut bacteria. Amoebiasis remains a significant but underreported global health issue, particularly in low-income countries [49]. Unlike obligate endosymbiotic systems, *Entamoeba* spp. interacts with a diverse gut microbiota, and these interactions can modulate disease severity. Co-infection studies have shown that enteric pathogens such as enteropathogenic *Escherichia coli* (EPEC) and *Salmonella typhimurium* can enhance *E. dispar* virulence [50,51]. In rodent models, co-infection with *S. typhimurium* led to increased inflammation and tissue damage, alongside the upregulation of virulence factors such as the Gal/GalNAc lectin, which promotes host cell adhesion [52]. Similar effects have been observed with EPEC, including the faster progression of amoebic liver abscesses [53]. However, it appears that these interactions are highly strain-specific. Different bacterial strains induce variable responses in *Entamoeba* spp., with some enhancing virulence expression and others having neutral or inhibitory effects [53,54]. Supporting this, exposure to *E. coli* has been shown to increase the motility of *E. histolytica*, a key virulence factor in tissue invasion and cytotoxicity, although the degree of this varies between strains [55] (Figure 3).

From a therapeutic perspective, amoebiasis is typically treated with metronidazole, but treatment failure and side effects are common [56]. The ability of gut bacteria to modulate parasite virulence suggests that the microbiome could influence treatment outcomes, e.g., bioactivation, biodegradation, and bioavailability, and therefore should be considered in terms of bioaccumulation within the bacteria or even competitive binding or the physical protection of the parasite by biofilm formation by the microbiota community [57]. Targeting these interactions would be challenging, as broad-spectrum antibiotics may disrupt beneficial microbiota, and strain-specific drugs reduce the feasibility of simple interventions. *Entamoeba*–bacteria interactions represent a context-dependent form of virulence modulation rather than a stable symbiotic dependency and highlight the importance of microbial context when considering parasite-targeted therapies.

## 6. Concluding Remarks

Endosymbiotic interactions represent a promising but fundamentally context-dependent target for antiparasitic therapy. The *Wolbachia*–filarial nematode system provides a good example of successful therapeutic exploitation, where obligate metabolic dependency enables the selective targeting of the parasite through the disruption of its bacterial symbiont. In contrast, such obligate relationships are rare among protozoan parasites, limiting the broader applicability of this approach. Instead, protozoan-associated bacteria more commonly engage in facultative interactions, as demonstrated in *Trichomonas vaginalis* and *Entamoeba* spp., where bacterial symbionts modulate virulence, immune responses and treatment outcomes rather than providing an essential function. These differences highlight that the utility of targeting symbiosis depends on several key factors, including the stability of the interaction, the degree of metabolic dependency and the accessibility of the symbiont to therapeutic agents.

Parasite–bacteria endosymbiosis is well documented and reviewed within the literature. This review compiled recent advances in the study of these symbiotic relationships, but it is important to note that this is not representative of the entire field, which is vast and spans multitudes of decades. Although symbiotic relationships may appear as an attractive therapeutic target, without extensive investigation into the complexity and essentiality of these symbiotic relationships, they will be challenging therapeutic targets; however, this review may help predict the results of polypathogen infections and aid in their management. Therefore, in the cases described above, improved testing for both specific organisms and the wider population would provide vital data to allow for progress in the understanding of symbiosis and disease outcomes to prevent a natural endogenous pool. This would avoid the “Trojan horse” effect, in which pathogenic bacteria are released within cells, causing untold harm and exacerbating the disease outcome, regardless of whether the parent parasite is lysed or not.

## 7. Outstanding Questions

What molecular mechanisms underpin the dependence of filarial nematodes on *Wolbachia* for reproduction and survival?

Why is obligate bacterial endosymbiosis rare in protozoan parasites compared to multicellular helminths?

Are there undiscovered obligate endosymbiotic relationships in clinically relevant protozoa that could be exploited therapeutically?

Should co-infections involving *Trichomonas vaginalis* and *Mycoplasma hominis* be routinely targeted in parallel during treatment?

To what extent can microbiome-based therapies complement or replace conventional antiparasitic treatments?

## Figures and Tables

**Table 1:** Examples of endosymbionts found in parasites.

Organism	Endosymbiont	Type of Relationship	Summary of Interaction	Effect of Therapeutic Intervention
**Nematodes;** *Onchocerca volvulus* *Dirofilaria* spp. *Wucheria bancrofti* *Brugia* spp.	*Wolbachia*	Obligate	Manipulation of host reproduction, riboflavin, haem, nucleotides and amino acids	Sterility of host females and death
*Trichomonas vaginalis*	*Mycoplasma hominis*	Facultative	Interdependent central metabolism, both cooperative (enhancing growth) and antagonistic (stimulating IFN)	Metronidazole treatments known to release Mycoplasma, a “Trojan horse” effect
**Enteropathogenic;** *Entamoeba histolytica Entamoeba dispar*	*Escherichia coli* *Salmonella typhimurium*	Bacterial endosymbiont but not essential for survival	Upregulation of virulence factors and lectins as adhesion molecules	Unclear, broad-spectrum antibiotics will affect beneficial microbiota
**SHTs;** *Strigomona culicis*, *Angomonas deanei* *Kentomonas* sp. *Novymonas esmeraldas*	*Candidatus* *Kinetoplastibacterium* sp. (beta-proteobacterium)	Bacterial endosymbiont essential for survival	Oxidative phosphorylation, haem, vitamins, amino acids	Treatment leads to death due to their intertwined metabolic dependencies

## Data Availability

No new data were created or analyzed in this study. Data sharing is not applicable to this article.

## Glossary

**Cytoplasmic incompatibility**	A type of reproductive manipulation caused by *Wolbachia* where infected males can only successfully reproduce with compatible infected females
**Endosymbiosis**	A form of symbiosis in which one organism lives within the cells or body of an organism
**Extracellular vesicles (EVs)**	Membrane-bound particles released by cells that can transfer molecules and modulate host immune responses
**Facultative symbiosis**	A non-essential interaction where organisms benefit from each other but can survive independently
**Filarial nematodes**	Parasitic worms that cause diseases such as lymphatic filariasis and onchocerciasis
**Helminths**	Multicellular parasitic worms
**Metabolic dependency**	A condition in which one organism relies on another for essential biochemical compounds it cannot produce itself
**Microbiome**	The community of microorganisms, including bacteria, that inhabit a particular environment, such as the human gut
**Obligate symbiosis**	A form of symbiosis in which at least one organism depends on the other for survival or reproduction
**Protozoa**	Single-celled eukaryotic organisms
**Symbiosis**	A close biological interaction between two different organisms, which may be beneficial, neutral or harmful
**Vector**	An organism that transmits a pathogen between hosts
**Virulence**	The degree to which a pathogen can cause disease in a host
***Wolbachia***	A genus of bacteria that infect arthropods and nematodes, forming obligate symbiotic relationships with them
